# The Role of miRNAs as Biomarkers for Pregnancy Outcomes: A Comprehensive Review

**DOI:** 10.1155/2017/8067972

**Published:** 2017-08-13

**Authors:** Martina Barchitta, Andrea Maugeri, Annalisa Quattrocchi, Ottavia Agrifoglio, Antonella Agodi

**Affiliations:** Department of Medical and Surgical Sciences and Advanced Technologies “GF Ingrassia”, University of Catania, Catania, Italy

## Abstract

Several studies showed that altered expression of the miRNA-ome in maternal circulation or in placental tissue may reflect not only gestational disorders, such as preeclampsia, spontaneous abortion, preterm birth, low birth weight, or macrosomia, but also prenatal exposure to environmental pollutants. Generally, the relationships between environmental exposure, changes in miRNA expression, and gestational disorders are explored separately, producing conflicting findings. However, validation of tissue-accessible biomarkers for the monitoring of adverse pregnancy outcomes needs a systematic methodological approach that takes also into account early-life environmental exposure. To achieve this goal, exposure to xenochemicals, endogenous agents, and diet should be assessed. This study has the aim to provide a comprehensive review on the role of miRNAs as potential biomarkers for adverse pregnancy outcomes and prenatal environmental exposure.

## 1. Introduction

MicroRNAs (miRNAs) are endogenous, short, noncoding molecules, which play a role in the mechanism of posttranscriptional gene expression by suppressing translation of protein-coding genes or cleaving target mRNAs [1–3]. A peculiar characteristic of miRNAs is represented by the fact that one miRNA can regulate the expression of several genes, while one gene can be targeted by different miRNAs [4], which means that miRNAs can regulate up to 30% of the human genome [3]. In fact, miRNAs represent important epigenetic mechanisms of regulation that can control complex processes such as cell growth, differentiation, stress response, and tissue remodeling, that, under particular conditions, can play a key role in many disease states [5, 6], including gestational disorders. In particular, miRNAs may reflect pathological gestational conditions [7], such as preeclampsia [8–10], spontaneous abortion [11–13], preterm birth [6, 14, 15], macrosomia [16, 17], or low birth weight [18]. Thus, their detection in maternal circulation makes miRNAs good candidate biomarkers to monitor the progression of normal pregnancy and the presence of gestational diseases [19], for the prevention and treatment of adverse pregnancy outcomes.

The aim of the present study was to provide a comprehensive review on the role of miRNA characterization as potential biomarkers for monitoring the most common adverse pregnancy outcomes with a focus on the influence of environmental exposure during pregnancy.

## 2. miRNAs and Preeclampsia

Preeclampsia (PE) is a leading global cause of maternal and perinatal mortality, affecting up to 8% of pregnancies [20]. PE is the result of impaired placental development and maladaptation to the gestational conditions which leads to clinical disturbs [21]. According to the clinical management guidelines for obstetrician-gynecologists, a pregnant woman can be diagnosed with PE when she suffers from blood pressure of 140 mm Hg systolic or higher or 90 mm Hg diastolic or higher that occurs after 20 weeks of gestation in a woman with previously normal blood pressure and proteinuria. PE can also be diagnosed in a severe form if the pregnant woman suffers from one of more of the following symptoms: blood pressure of 160 mm Hg systolic or higher or 110 mm Hg diastolic or higher on two occasions at least 6 hours apart while the patient is on bed rest; proteinuria of 5 g or higher in a 24-hour urine specimen or 3+ or greater on two random urine samples collected at least 4 hours apart; oliguria of less than 500 mL in 24 hours; cerebral or visual disturbances; pulmonary edema or cyanosis; epigastric or right upper-quadrant pain; impaired liver function; thrombocytopenia; and fetal growth restriction [22]. In order to prevent severe consequences for both the mother and the fetus, it is essential to identify women at risk of PE at an early stage [20]. In this scenario, much progress has been made to characterize miRNAs as potential biomarkers able to identify women at risk of PE. Up to date, several studies described the differential regulation of miRNAs in pregnant women suffering from PE and in healthy controls (Table 1). In 2012, Wu and collaborators detected 15 differentially expressed miRNAs in serum of severe preeclamptic women and healthy controls: miR-574-5p, miR-26a, miR-151-3p, miR-130a, miR-181a, miR-130b, miR-30d, miR-145, miR-103, miR-425, miR-221, miR-342-3p, and miR-24 were upregulated, while miR-144 and miR-16 were downregulated in PE women. Among these fifteen miRNAs, seven were further confirmed as upregulated in PE women by quantitative RT-PCR (miR-24, miR-26a, miR-103, miR-130b, miR-181a, miR-342-3p, and miR-574-5p; fold change, FC = 1.89–3.77) [23]. miR-26a and miR-342-3p were also detected as upregulated in PE women by Choi and colleagues, who isolated from formalin-fixed and paraffin-embedded samples of the placenta thirteen miRNAs (miR-92b, miR-197, miR-342-3p, miR-296-5p, miR-26b, miR-25, miR-296-3p, miR-26a, miR-198, miR-202, miR-191, miR-95, and miR-204) significantly overexpressed (FC = 2.03–12.28). Conversely, they detected miR-21 and miR-223 as underexpressed in women suffering from severe PE (FCs = 0.33 and 0.40, resp.) [8]. A study by Ura et al. detected 19 differentially expressed miRNAs in severe PE women: 12 were upregulated (miR-1233, miR-650, miR-520a, miR-215, miR-210, miR-25, miR-518b, miR-193a-3p, miR-32, miR-204, miR-296-5p, and miR-152), while 7 were downregulated (miR-126, miR-335, miR-144, miR-204, miR-668, miR-376a, and miR-15b). The results obtained by TaqMan array analysis were validated through quantitative real-time PCR, that confirmed that severe PE is associated with the upregulation of miR-1233, miR-520, and miR-210 (FC = 3.1–5.4) and the downregulation of miR-144 (FC = 0.39) during the early stages of pregnancy [24].

In 2013, a two-step study identified 51 miRNAs differentially expressed in women suffering from severe or mild PE compared to healthy pregnant women. The first step used a sequencing method to identify 22 upregulated miRNAs and 5 downregulated miRNAs in plasma of women suffering from severe PE. Furthermore, the researchers showed that 33 miRNAs were upregulated and 6 miRNAs were downregulated in plasma of mild preeclamptic women. The second step of the study focused on four different miRNAs (miR-141, miR-144, miR-221, and miR-29a) selected among the 51 previously detected, in order to validate the results on a larger number of samples through a RT-PCR method. The results from this second step confirmed the upregulation of miR-141, miR-221, and miR-29a in women suffering from severe PE and the upregulation of miR-141 and miR-29a and the downregulation of miR-144 in women suffering with mild PE [9]. In a retrospective study by Munaut and colleagues, four different miRNAs (miR-210-3p, miR-210-5p, miR-1233-3p, and miR-574-5p) were identified as upregulated in serum of PE women [10], while Sandrim and collaborators found miR-885-5p significantly overexpressed in plasma of PE women (FC = 4.5) [25].

In a recent study by Hromadnikova and colleagues, the differential expression pattern of C19MC miRNAs was detected in plasma of preeclamptic women compared to healthy controls. Particularly, levels of miR-517-5p, miR-518b, and miR-520h increased during the first trimester of gestation in women who developed PE (FC = 3.1–8.9) [26].

## 3. miRNAs and Spontaneous Abortion

Abortion, defined by the World Health Organization (WHO) as “any interruption of pregnancy before 28 weeks of gestation with a dead fetus” [27], is the most common complication in human reproduction, with an incidence ranging from 50 to 70% of all conceptions [28]. The spontaneous interruption of two or more pregnancies consists of a different disorder, which is the recurrent pregnancy loss (RPL), that affects 5% of pregnant women suffering from this disease [29]. Although the known causes of RPL are cytogenetic abnormalities, antiphospholipid syndrome, uterine anomalies, hereditary thrombophilia, autoimmunity, sperm quality, and environmental factors, it is not possible to understand its etiology in most of the cases [30]. Unexplained recurrent pregnancy loss (URPL) is a major challenge in the obstetric field as it lacks of both safe and effective therapies and reliable methods of early diagnosis [13].

In the uterus, miRNAs can regulate the expression of genes associated with the anti-inflammatory response at the time of peri-implantation and can also have a role in the maternal-fetal immune tolerance [30].  Table 2 shows the results of studies that have investigated the role of miRNA regulation in RPL; among these, a study conducted on 12 childless Chinese women with three or more spontaneous miscarriages at the 7th week of gestation and on women with induced abortion, showed that miR-133a was highly overexpressed in the villi of the RPL cases (FC = 32.4) [31]. Furthermore, JEG-3 cell lines were cultured and transfected with pre-miR-133a. Luciferase reporter assays and subsequent Western blot analysis showed that cell lines transfected with pre-miR-133a had a decreased HLA-G expression when compared with control cell lines [31]. HLA-G is a nonclassical major histocompatibility complex which is expressed in the placenta during the full length of gestation and almost solely in the extravillous trophoblasts at the fetal-maternal interface [32]. The peculiar localization of HLA-G suggests that it plays a crucial role in the maternal immune tolerance to the fetus [31]; however, it is known that its expression is associated with spontaneous abortion [33]. Thus, the study by Wang and colleagues provides evidences that miR-133a is involved in the pathogenesis of RPL by reducing the translation of HLA-G by binding its 3′-UTR [31].

Further analysis on the identification of miRNA profiles in villi of RPL cases has been conducted by Dong and colleagues in 2014. In their study, differences in miRNA expression were observed in villus tissues and decidua tissues obtained from 20 Chinese women suffering from RPL, compared to those in 15 clinically normal controls. In the villus of RPL women, 41 miRNAs were found as downregulated, whereas four were upregulated. In the decidua of RPL women, seven miRNAs were overexpressed. However, to further filter the key miRNAs involved in the RPL processes, new fold change criteria were selected (≥2 or ≤0.2): miR184, miR187, and miR125b-2 were significantly overexpressed in the villus of RPL women, whereas miR520f, miR3175, and miR4672 were downregulated. As far as the decidua is concerned, miR517c, miR591a-1, miR522, miR520h, and miR184 were found upregulated in RPL women, compared to those in normal controls. High levels of miR184 were found in both samples, suggesting that it is involved in RPL [34].

In 2016, Li and Li isolated NK (Natural Killer) cells from decidua tissue of both women suffering from URPL and healthy controls and identified six differentially expressed miRNAs: miR-34a, miR-155, miR-141, miR-125a, and miR-125b were upregulated (FC = 1.85–3.96), while miR-24 was downregulated, in the URPL group, compared with the those in healthy controls (FC = 0.64) [11]. Interestingly, members of the miR-24 family are regulators of p53 and thus involved in the mechanisms of apoptosis and cell proliferation [35]; however, this was the first study showing a possible association with RPL [11].

Further studies on URPL have been conducted by Qin and colleagues, who screened circulating miRNAs isolated from plasma of women suffering from URPL and healthy controls. Their findings showed that 6 miRNAs had differential expression between cases and controls and thus could serve as a potential candidate biomarker for URPL. Particularly, miR320b, miR146b-5p, miR221-3p, and miR559 were upregulated (FC = 3.06–4.79) whereas miR101-3p was downregulated (FC = 0.21). However, the use of miRNAs as noninvasive diagnostic biomarkers has not been established yet and the sample size of the study was small. Thus, studies on larger populations are needed in order to validate the potential role of these miRNAs as biomarkers for URPL [13].

## 4. miRNAs and Preterm Birth

Preterm birth (PTB) is defined as birth occurring earlier than the 37th week of gestation or before 259 days since the first day of a woman's last menstrual period [36]. Premature birth, especially very premature birth, is a major cause of neonatal mortality, morbidity, disability [37], and cognitive impairment, in both childhood and adolescence [38]. In fact, complications of preterm birth are the single largest direct cause of neonatal deaths, responsible alone for 35% of the world's 3.1 million deaths a year, and the second most common cause of under 5-year-old deaths after pneumonia [39]. Although risk factors for PTB such as inflammation [40] and cervical length [41] are known, the major obstacle to the assessment of impactful interventional strategies against PTB is represented by the lack of understanding of the critical molecular mechanisms involved in its pathogenesis [14].

Epigenetic dysregulation may contribute to a substantial part of the risk of PTB [42], and miRNA modulation may represent an epigenetic mechanism that may be both a biomarker of risk and a target potentially amenable to future interventions [15]. Hassan and colleagues provided the first evaluation of a differential miRNA expression in cervical tissue of women undergoing term labor and delivery: in fact, among the 226 miRNA expressed in the cervical tissue, three (miR-223, miR-34b, and miR-34c) were differentially expressed and upregulated, compared to woman not in labor. However, the study wasn't able to investigate on the changes of the cervix during the progression of the pregnancy [43]. The process of connective tissue remodeling in the cervix, during pregnancy, occurs in four stages (softening, ripening, dilation, and repair), which are overlapping in time but uniquely regulated [44]. Elovitz and collaborators gave a substantial contribution on this topic, setting up a “RNA PAP” method that demonstrates that a noninvasive molecular assessment of human cervix during pregnancy is feasible. The “RNA PAP” technique is like a Pap smear and involves the collection of ectocervical cells through a cytobrush that can be performed in each trimester of pregnancy. The molecular analysis on the miRNA-ome, conducted on samples collected with this method, showed that miRNA profiles in cervical cells may distinguish women who are at risk for PTB. Among the 99 differentially expressed miRNA between women undergoing PTB and controls, 24 had a >2-fold change in expression. Among these, only two (miR-143 and miR-145) were increased in women who eventually had a PTB (FC = 11.5 and 12.34, resp.) and both were negatively correlated to cervical length. [14]. Further studies, conducted by Elovitz and colleagues on miRNA isolated from serum of pregnant women, showed an alteration in the structure of four different miRNAs (miR-200a, miR-4695-5P, miR-665, and miR887) between PTB women and controls: miR-200a and miR-4695-5P were in their mature form, whereas miR-665 and miR887 were in their nonactive stem-loop form. However, miRNA profiles in the maternal blood were not significantly different in women who were destined to have a preterm, compared with a term birth. The results are in contrast with previous findings but may suggest that PTB is a “local” disturb, with molecular and cellular changes at the level of the reproductive tissues [6]. The role of local miRNAs in reproductive tissues might help to significantly advance understanding of preterm birth. Sanders et al. showed that six miRNAs (miR-21, miR-30e, miR-142, miR-148b, miR-29b, and miR-223), isolated from cervical cells, were significantly overexpressed in women who had a shorter gestation. Interestingly, three of these miRNAs (miR-21, miR-142, and miR-223) have not been isolated in any previous studies concerning PTB or gestational age in general [15].  Table 3 summarizes the miRNAs involved in the etiology of PTB.

## 5. miRNAs and Birth Weight

The WHO has defined “low birth weight” (LBW) as a weight at birth of less than 2500 grams [45]. Infants' LBW can be caused both by PTB or restricted fetal intrauterine growth [46], and it is a risk factor for fetal and neonatal mortality, growth, and cognitive development inhibition and chronic diseases in adult life [47]. On the other hand, macrosomia has been defined by the American College of Obstetricians and Gynecologists as a weight at birth over 4000 grams, regardless of the gestational age or greater than the 90th percentile for gestational age, adjusting for neonatal sex and ethnicity [48]. Maternal overweight and metabolic disorders such as diabetes mellitus type 2 or gestational diabetes mellitus play a key role in macrosomia [49] which is known since the '80s for leading to complications such as prenatal asphyxia, trauma, and fetal death [50]. Several studies have been conducted in order to find an association between miRNAs and these birth outcomes with the aim to establish biomarkers useful for the diagnosis of these disorders (Table 4). As far as LBW is concerned, Song and colleagues studied the expression of placenta-specific miR-517a in placental tissue and maternal serum. The expression of mir-517a was higher both in the placenta of LBW infants (FC = 12.33) and in the serum of women who delivered a LBW baby (FC = 8.03). Further analysis conducted on cultured cells suggested that mir-517a inhibits trophoblast invasion, leading to abnormal placentation and, thus, to LBW [18]. Further studies, conducted on placenta-specific miRNAs, showed that the expression of miR-518b and miR-519a was altered in infants with IUGR, when compared to large for gestational age (LGA) infants and healthy babies: specifically, miR-518b was down regulated (FC = 0.46), whereas miR-519a was up regulated (FC = 1.91) [51]. miR-518b and the other C19MC miRNAs were recently studied also by Hromadnikova and colleagues, who screened first the trimester blood of IUGR subjects and healthy controls. However, no significant difference in plasma levels of C19MC miRNAs was found between the two groups [26]. Researches on macrosomia are more numerous. In 2014, a two-phase study conducted on 120 participants showed aberrant expression of miR37a, which was downregulated in serum of pregnant women who delivered infants with macrosomia [16]. In 2015, Li and colleagues investigated on the role of miRNA17-92 cluster in macrosomia, with a study on both the placenta and serum from 57 mothers who delivered infants with macrosomia and 100 healthy controls. The results on placenta showed the upregulation of miR17, miR18a, miR19a, miR19b, miR20a, and miR92a in samples coming from mothers with macrosomic babies. As far as the maternal serum is concerned, miR17, miR18a, miR19a, and miR92a were downregulated in plasma of mothers with macrosomic infants. This result has high diagnostic sensitivity and specificity for macrosomia, as suggested by the ROC curve analysis [52]. Further studies on miRNA profile in serum of pregnant women were conducted by Ge and collaborators, investigating 45 pregnant women who eventually delivered infants with macrosomia and 30 women who eventually delivered healthy infants. Firstly, samples of women with fetal macrosomia and women with a healthy pregnancy were analysed through TaqMan low-density array (TLDA). Particularly, 143 miRNAs were found differentially expressed: 43 were up regulated and 100 were down regulated in women with fetal macrosomia. Among these, 12 miRNAs were chosen for the validation through qRT-PCR. Four out of the 12 selected miRNAs were upregulated (miR-661, miR-523-3p, miR-125a-5p, and miR-30a-3p) while 8 were downregulated (miR-181a-5p, miR-200c-3p, miR-143-3p, miR-221-3p, miR-16-5p, miR-141-3p, miR-18a-5p, and miR-451). All the results by TLDA were confirmed by qRT-PCR with the exception of miR-16-5p that had opposite results. ROC curve analysis was performed in order to test the characteristic of the differentially expressed miRNAs. Downregulated miR-523-3p, miR200c-3p, and miR141-3p showed a higher efficiency in distinguishing between woman with fetal macrosomia and women with normal pregnancy; thus, further analysis through qRT-PCR was conducted in order to verify their specificity for macrosomia. In fact, further analysis on serum of 16 pregnant women suffering from PE showed that miR141-3p and miR200c-3p were downregulated in women with fetal macrosomia, also when compared to those in preeclamptic woman, whereas miR-523-3p did not show a significant difference between the two groups [53]. In 2016, a study on placental expressed miRNAs on 67 samples of macrosomic placental tissues and 64 normal placental tissues showed that high levels of miR-21 and low levels of miR-143 are associated with macrosomia [54]. MiR-21 was also found associated to macrosomia by Jiang and colleagues; however, findings of this study on maternal serum showed that levels of miR-21 were significantly lower in serum of women delivering macrosomic infants when compared to healthy controls [17]. Miura and collaborators found an association between circulating miR-21 levels in maternal serum and not only infants' birth weight but also maternal body mass index. However, no significant association was found between circulating miR-21 levels and placental weight [55].

## 6. miRNAs and Environmental Exposure during Pregnancy

Studies on birth cohorts using miRNAs as biomarkers in pregnancy have been mostly conducted to assess the association between the exposure to environmental pollutants and changes in the miRNA-ome in placental samples (Table 5). A study on placental samples from the National Children's Study (NCS) Vanguard birth cohort investigated on the association between miRNA profile in placentas and prenatal exposure to dichlorodiphenyldichloroethylene (DDE), bisphenol A (BPA), polybrominated diphenyl ethers (PBDEs), polychlorinated biphenyls (PCBs), arsenic (As), mercury (Hg), lead (Pb), and cadmium (Cd). Results showed that exposure to high levels of Hg was associated to decreased levels of miR-151p, miR-10a, miR-193b, miR-1975, miR-423-5p, miR-520d-3p, miR-96, miR-252a, miR-518d-5p, miR-520a-5p, miR-190, and numerous miRNAs belonging to the let-7 family. Exposure to high levels of Pb was associated not only to lower levels of the let-7 family miRNAs and miR-190 but also to lower levels of miR-146a, miR-10a, and miR-431, while the levels of miR-651 were increased. Exposure to high levels of Cd and PCBs were associated with high levels of miR-1537. However, the study did not take into account birth outcomes and analysed only 110 placentas collected from the larger NCS Vanguard birth cohort [56].

In 2016, a study analysed sample tissues of placenta from the ENVIR*ON*AGE birth cohort to assess a possible association between exposure to air pollutants (PM_2.5_ and NO_2_) and altered expression of placental miRNAs. As far as PM_2.5_ is concerned, exposure to the pollutants in the first trimester of pregnancy was positively associated with increased expression of miR-20a and miR-21, while exposure during the second trimester was associated with lower expression of miR-16, miR-20a, miR-21a, miR-222, and miR146a, which levels were even lower during the third trimester. Exposure to NO_2_ during the first trimester was positively associated with an increased expression of miR-21, whereas the exposure to the pollutant during the second trimester of pregnancy showed an association with lower levels of miR-20a, miR-21, and miR-146a. Although this study has a larger sample size (210 mother-newborns pairs) than the other previously described, it does not take into account pregnancy outcomes [57].

A study conducted on 179 samples of urines and placentas of first-trimester mothers and their infants, coenrolled in the Harvard Epigenetic Birth Cohort and in the Predictors of Preeclampsia Study, investigated on the alteration of placental miRNA expression and exposure to phthalate metabolites and phenol. After a pilot study on 48 samples, LaRocca and colleagues selected 29 differentially expressed miRNAs to be further investigated in the full study. The most relevant results showed that increased levels of total phenols in the urine were associated with lower expression of miR-142. Particularly, increased levels of parabens were associated with lower expression of miR-142 both in urine and in the placenta. Increasing levels of nonparabens in urine was also associated to lower levels of miR-15a-5p. However, only in female newborn placentas, the expression levels of miR-15a-5p were found significantly associated to an increasing exposure to total phenols. As far as the phenols were concerned, the expression of the majority of tested miRNAs was positively associated to levels of BPA and BP-3 in urines. Results about phthalates showed that an increased exposure to low molecular weight phthalates was associated to lower levels of miR-185 expression in placenta. The result was probably due to MEP (monoethyl phthalate), which was the compound presenting the stronger association with miR-185. Moreover, the expression of the majority of the tested miRNAs was associated to the exposure to monocarboxyisooctyl phthalate (MOCP). However, no association between the expression of the miRNAs altered by the maternal chemical exposure and gestational age, birth weight, or birth length was found [58].

## 7. Discussion

Several studies have investigated on the association between circulating levels of miRNAs and birth outcomes, to establish potential biomarkers for the prevention of the most common gestational disorders. In fact, the aforementioned scientific literature well describes aberrant miRNA expression in mother-child pairs with adverse pregnancy outcomes, suggesting that miRNAs may serve as useful prevention and clinical tools.

However, results from several works are inconsistent and the spectrums of miRNAs observed by different studies are rather controversial. Such inconsistency or discrepancies can be attributed to differences in sample type, sample handling, gestational age at sampling, techniques used for miRNA profiling, and population characteristics, such as age, ethnicity, and lifestyles.

Particularly, the results could be influenced by the different sources of miRNAs, such as the placenta, umbilical cord blood, or maternal sera. In biomarker-related studies, both these noninvasive and easily obtainable samples are suitable sources of miRNAs, reflecting early-life experience during pregnancy. However, changes in miRNA expression, using the cord blood and placenta as starting material, could be analysed to understand the etiology of adverse pregnancy outcomes, because the obtained expression profiles reflect the environmental exposure toward the end of pregnancy.

To investigate the relationship between placental-specific miRNAs and pregnancy outcomes, researchers compared the expression of miRNAs isolated from the placentas between patients affected by the disorders and healthy controls [8, 51, 54, 56–58]. Since placental tissues are obtained at the time of delivery, it is unclear whether the aberrant miRNA expression is the cause or the consequence of the disorder. Accordingly, only two studies analysed the expression of specific miRNAs both in the placenta and maternal serum [18, 52].

In fact, maternal plasma and serum could be useful to monitor changes in miRNA expression throughout pregnancy and in specific gestational periods, allowing the development of screening biomarkers. Other sample sources were maternal urine [58], decidua [11, 34], villi [31, 34], and cervical cells [14, 15].

Blood-derived biomarkers could be routinely monitored, representing the preferred specimens for noninvasive diagnosis. However, gestational age at sampling could influence the levels of circulating miRNAs. Among the reviewed studies, gestational age at sampling depended on the study design and sample type, with a range from 7 to 40 weeks. As a result, it is discouraged to compare findings from studies which evaluate miRNA expression levels at different gestational age. To overcome this issue, studies have to include results for each trimester of the pregnancy, as provided by Tsamou et al. and LaRocca et al. [57, 58].

Endogenous degrading properties, elapsed time from collection, temperature during transport, anticoagulant, and stabilising agents are key factors that affect the quality of biological samples and expression analyses. However, among the reviewed studies, it is unclear whether sample processing and storage were appropriate.

To date, there are several methods to examine miRNA profiling, such as quantitative real-time PCR (qRT-PCR), microarrays, and direct sequencing. Although each method has advantages and limitations, qRT-PCR seems to have better sensitivity than array technologies for miRNA profiling [59]. On the other hand, qRT-PCR approach is limited in the number of detectable miRNAs compared with microarrays. Next-generation sequencing (NGS) offers important advantages over other technologies, representing the best platform for miRNA discovery. However, although NGS is extremely sensitive, qRT-PCR is still the only platform capable of generating absolute quantification [59]. Differences in techniques used for miRNA profiling are also a contributing factor controversial in the specific miRNA expression. The suitable approach, mostly used, was to identify disease-related differences in miRNA expression by miRNA microarray or second-generation sequencing in small subgroups, followed by the validation by qRT-PCR on a larger sample size [8, 9, 13, 14, 16, 23–25, 31, 34, 53, 58]. However, other studies just used qRT-PCR techniques to validate candidate biomarkers identified through literature search or based on previous findings [10, 11, 18, 26, 51, 52, 54, 55, 57]. In addition, variability between studies was also observed in data reporting. In fact, the majority of studies used 2-fold change and 0.5-fold change thresholds to define marked differential miRNA expression [8, 13, 14, 18, 24, 25, 31]. However, others reported as differentially expressed also miRNA with an expression of only 1.5-fold changes [11, 23, 51]. Thus, to improve accuracy and avoid discrepancies, it is important to develop standardized methodologies both in preanalytical and analytical activities.

An additional limitation of studies mentioned in this paper was the size of the enrolled population. Although several evidences demonstrate the potential application of miRNAs in the monitoring of adverse pregnancy outcomes, data interpretation must be prudent because of the small sample size.

In this context, a major involvement of large birth cohorts and biobanks is warranted.

Moreover, the validation of tissue-accessible biomarkers for the monitoring of adverse pregnancy outcomes needs a systematic approach that takes also into account early-life environmental exposure [60]. To achieve this goal, exposure to xenochemicals, endogenous agents, and diet should be assessed through the collection of questionnaire data, as well as biological samples.

At the best of our knowledge, few studies have investigated miRNAs involved in causal pathways between early-life exposure and adverse pregnancy outcomes [58]. Generally, the relationships between environmental exposure, changes in miRNA expression, and gestational disorders are explored separately, producing conflicting findings due to methodological and biospecimen heterogeneity.

## 8. Conclusion

The overall aim of this review was to summarize the effects of miRNAs and environmental exposure on pregnancy outcomes. Although several studies reported differential miRNA expression in gestational disorders, we arose the need of more standardized methodologies in both preanalytical and analytical levels, as well as a major involvement of large birth cohorts and biobanks. Particularly, the potential to transform biobanks into well-characterized longitudinal epidemiological studies has become crucial to the conduct of large-scale genomic and epigenomic researches. The set of exposure information, clinical data, and biological samples, collected in human biobanks, constitutes a resource to conduct further analyses for the characterization and validation of miRNAs as biomarkers in both preventive and diagnostic strategies for gestational disorders.

## Figures and Tables

**Table 1:** Studies reporting an association between alteration of miRNA expression and PE.

Authors and year	Study design	Enrolled population	Samples	Gestational age at sampling (weeks)	Techniques	↑ miRNAs	↓ miRNAs
Wu et al., 2012 [23]	Retrospective	10 cases + 10 controls	Maternal serum	37–40	miRNA microarray + qRT-PCR	miR-24, miR-26a, miR-103, miR-130b, miR-181a, miR-342-3p, and miR-574-5p (cases)	
Choi et al., 2013 [8]	Retrospective	21 cases + 20 controls	Placental tissue	35–40	miRNA microarray + qRT-PCR	miR-24, miR-26a, miR-103, miR-130b, miR-181a, miR-342-3p, and miR-574-5p (cases)	miR-21 and miR-223 (cases)
Ura et al., 2014 [24]	Retrospective	24 cases + 24 controls	Maternal serum	12–14	TLDA + qRT-PCR	miR-1233, miR-520, miR-210 (cases)	miR-144 (cases)
Li et al., 2013 [9]	Retrospective	*First step*:4 mild PE + 4 severe PE + 4 controls	Maternal serum	32–38	SOLiD sequencing	miR-519d, miR-517b, miR-517c, miR-26b, miR-221, miR-521, miR-378, miR-519a, miR-520h, miR-125b, miR-29a, miR-125a-5p, miR-114, miR-30a, miR-518c, miR-27a, miR-519e, miR-130a, miR-515-3p, miR-299a-5p, miR-518b, miR-23a, miR-23b, miR-34a, miR-424, miR-525-3p, miR-199a-5p, miR-29b, miR-99a, miR-21, miR-145, miR-512-5p, and miR-30b (mild and severe PE)	miR-15b, miR-223, miR-320c, miR-185, miR-107, miR-451, and let-7f (mild and severe PE)
						miR-19a, miR-10a, miR-144, miR-151b, miR-144, miR-182, and miR-19b (severe PE)	miR-19a, miR-144, miR-19b, and miR-25 (mild PE)
		*Validation step*: 16 mild PE + 22 severe PE + 32 controls		28–38	qRT-PCR	miR-141, miR-29a, (mild PE)	miR-144 (mild PE)
						miR-141, miR-221, and miR-29a (severe PE)	
Munaut et al., 2016 [10]	Retrospective	23 cases + 44 controls	Maternal serum	31-32	qRT-PCR	miR-210-3p, miR-210-5p, miR-1233-3p, and miR-574-5p (PE women)	**—**
Sandrim et al., 2016 [25]	Retrospective	*Screening*: 5 cases + 4 controls	Maternal plasma	35	PCR array + qRT-PCR	miR-885-5p (PE women)	miR-376c-3p, miR-19a-3p, and miR-19b-3p (PE women)
		*Case control study*: 19 cases + 14 controls		35		miR-885-5p (PE women)	—
		*Replication study*: 8 cases + 8 controls		35		miR-885-5p (PE women)	—
Hromadnikova et al., 2017 [26]	Retrospective	21 PE + 58 controls	Maternal plasma	10-11	qRT-PCR	miR-517-5p, miR-518b, and miR-520h (PE women)	**—**

qRT-PCR: quantitative real-time polymerase chain reaction; TLDA: TaqMan low-density array; PE: preeclampsia; IUGR: intrauterine growth restriction.

**Table 2:** Studies reporting an association between alteration of miRNA expression and spontaneous abortion.

Authors and year	Study design	Enrolled population	Samples	Gestational age at sampling (weeks)	Techniques	↑ miRNAs	↓ miRNAs
Wang et al., 2012 [31]	Retrospective	12 cases + 10 controls	Villi	7	Microarray + qRT-PCR	miR-133a	
Dong et al., 2014 [34]	Retrospective	20 cases + 15 controls	Villi and decidua	7	Microarray + qRT-PCR	miR-184, miR-187, and miR-125b-2 (villi RPL)—miR-517c, miR-519a-1, miR-522, miR-520h, miR-184 (decidua RPL)	miR-520f, miR-3175, and miR-4672 (villi RPL)
Li and Li., 2016 [11]	Retrospective	20 cases + 20 controls	Decidua	7–10	qRT-PCR	miR-34a, miR-155, miR-141, miR-125a, and miR-125b (RPL)	miR-24 (RPL)
Qin et al., 2016 [13]	Retrospective	27 cases + 28 controls	Maternal plasma	7	miRNA array + qRT-PCR	miR-320b, miR-146b-5p, miR-221-3p, and miR-559 (cases)	miR-101-3p (cases)

qRT-PCR: quantitative real-time polymerase chain reaction; RPL: recurrent pregnancy loss.

**Table 3:** Studies reporting an association between alteration of miRNA expression and PTB.

Authors and year	Study design	Enrolled population	Samples	Gestational age at sampling (weeks)	Techniques	↑ miRNAs	↓ miRNAs
Elovitz et al., 2014 [14]	Retrospective	10 cases + 10 controls	Cervical cells	<37	Affymetrix GeneChip miRNA array + qRT-PCR	miR-143, miR-145 (cases)	—
Elovitz et al., 2015 [6]	Retrospective	40 cases + 40 controls	Maternal serum	<30	miRNA array	miR-4695-5P, miR-665 (stem-loop), and miR-887 (stem-loop) (cases)	miR-200a (cases)
Sanders et al., 2016 [15]	Prospective	60 pregnant women, 4 lost at follow-up	Cervical cells	<37	NanoString	miR-21, miR-30e, miR-142, miR-148b, miR-29b, and miR-223 (cases)	

qRT-PCR: quantitative real-time polymerase chain reaction.

**Table 4:** Studies reporting an association between alteration of miRNA expression and altered BW.

Authors and year	Gestational disorder	Study design	Enrolled population	Samples	Gestational age at sampling (weeks)	Techniques	↑ miRNAs	↓ miRNAs
Song et al., 2013 [18]	LBW	Retrospective	10 cases + 20 controls	Placental tissue + maternal serum	Delivery	qRT-PCR	miR-517a (maternal serum and placental tissue of cases)	
Wang et al., 2014 [51]	LGA, IUGR	Retrospective	30 LGA + 30 IUGR + 30 controls	Placental tissue	Delivery	qRT-PCR	mirR-519a (IUGR)	miR-518b (IUGR)
Wang et al., 2014 [51]	LGA	Retrospective	30 LGA + 30 IUGR + 30 controls	Placental tissue	Delivery	qRT-PCR	—	—
Hromadnikova et al., 2017 [26]	IUGR	Retrospective	18 IUGR + 58 controls	Maternal plasma	10	qRT-PCR	—	**—**
Hu et al., 2014 [16]	Macrosomia	Retrospective	60 cases + 60 controls	Maternal serum	16–20	TLDA − qRT-PCR	—	miR-376a
Li et al., 2015 [52]	Macrosomia	Retrospective	57 cases + 100 controls	Placental tissue + maternal serum	Placenta (delivery) serum (NA)	qRT-PCR	miR-18a, miR-19a, miR-20a, miR-19b, and miR-92a (placental tissue of cases)	miR17, miR18a, miR19a, and miR92a (maternal serum of cases)
Ge et al., 2015 [53]	Macrosomia	Retrospective	35 cases + 20 controls	Maternal serum	18–28	TLDA + qRT-CR	miR-523-3p, miR-3a-3p, and miR-16-5p (cases)	miR-221-3p, miR-143-3p, miR-18a-5p miR-141-3p, and miR200c-3p (cases)
Zhang et al., 2016 [54]	Macrosomia	Retrospective	67 cases + 64 controls	Placental tissue	Delivery	qRT-PCR	miR-21	miR-143
Jiang et al., 2015 [17]	Macrosomia	Retrospective	60 cases + 60 controls	Maternal plasma	16–20 weeks and 1 week from delivery	TLDA + qRT-PCR	—	miR-21
Miura et al., 2015 [55]	Birth weight	Cross-sectional	82 pregnant women	Maternal serum	37-38	qRT-PCR		

LBW: low birth weight; qRT-PCR: quantitative real-time polymerase chain reaction; IUGR: intrauterine growth restriction; LGA: large for gestational age; TLDA: TaqMan low-density array.

**Table 5:** Studies reporting an association between alteration of miRNA expression and environmental exposure.

Authors and year	Exposure	Study design	Enrolled population	Samples	Techniques	↑ miRNAs	↓ miRNAs
Li et al., 2015 [56]	Organic and inorganic environmental pollutants	Prospective	110 clinical normal pregnant women	Placental tissue	NanoString	miR-651 (Pb exposure) miR-1537 (Cd exposure) miR-188-5p (PBDE high brominated congener 209 exposure) miR-1537 (PCB congener 52 and 10 and total PCB exposure)	miR-151p, miR-10a, miR-193b, miR-1975, miR-423-5p, miR-520d-3p, miR-96, miR-252a, miR-518d-5p, miR-520a-5p, miR-190, let-7a, let-7b, let-7c, let-7d, let-7g, and let-7i (Hg exposure) let-7f, miR-146a, miR-10a, and miR-431 (Pb exposure) let-7c (PBDE low brominated congener 99 exposure)
Tsamou et al., 2016 [57]	PM_2.5_, NO₂	Prospective	210 mother-newborn pairs	Placental tissue	qRT-PCR	*1st trimester* miR-20a, miR-21 (PM_2.5_ exposure) miR-21 (NO₂ exposure)	*1st trimester* —
						*2nd trimester* **—**	*2nd trimester* miR-16, miR-21, miR-146a, and miR-222 (PM_2.5_ exposure) miR-20a, miR-21, and miR-146a (NO₂ exposure)
						*3rd trimester* **—**	*3rd trimester* miR-146a, (PM_2.5_ exposure)
LaRocca et al., 2016 [58]	Endocrine Disrupting Chemicals	Prospective	179 women	Maternal urine + placental tissue	PCR Array + qRT-PCR	mir-15a-5p (Σ^∗^* phenols*, Σ *parabens* male placentas) mir-185 (Σ *DEHPm*, Σ *LMW*)	mir-15a-5p (Σ *phenols*, Σ *parabens* female placentas and *nonparabens*, overall) miR-142-3p (Σ *phenols*, Σ *parabens*, Σ *nonparabens* overall) mir-185 (Σ *phthalates*, Σ *HMW*, overall)

qRT-PCR: quantitative real-time polymerase chain reaction; ∗ indicates the sum of all the chemicals.

## References

[1] Montagnana M., Danese E., Lippi G., Fava C. (2017). A narrative review about blood laboratory testing for early prediction of preeclampsia: chasing the finish line or at the starting blocks?. *Annals of Medicine*.

[2] Guo H., Ingolia N. T., Weissman J. S., Bartel D. P. (2010). Mammalian microRNAs predominantly act to decrease target mRNA levels. *Nature*.

[3] Baek D., Villen J., Shin C., Camargo F. D., Gygi S. P., Bartel D. P. (2008). The impact of microRNAs on protein output. *Nature*.

[4] Sayed D., Abdellatif M. (2011). MicroRNAs in development and disease. *Physiological Reviews*.

[5] Bartel D. P. (2004). MicroRNAs: genomics, biogenesis, mechanism, and function. *Cell*.

[6] Elovitz M. A., Anton L., Bastek J., Brown A. G. (2015). Can microRNA profiling in maternal blood identify women at risk for preterm birth?. *American Journal of Obstetrics and Gynecology*.

[7] Prieto D. M. M., Markert U. R. (2011). MicroRNAs in pregnancy. *Journal of Reproductive Immunology*.

[8] Choi S. Y., Yun J., Lee O. J. (2013). MicroRNA expression profiles in placenta with severe preeclampsia using a PNA-based microarray. *Placenta*.

[9] Li H., Ge Q., Guo L., Lu Z. (2013). Maternal plasma miRNAs expression in preeclamptic pregnancies. *BioMed Research International*.

[10] Munaut C., Tebache L., Blacher S., Noël A., Nisolle M., Chantraine F. (2016). Dysregulated circulating miRNAs in preeclampsia. *Biomedical Reports*.

[11] Li D., Li J. (2016). Association of miR-34a-3p/5p, miR-141-3p/5p, and miR-24 in decidual natural killer cells with unexplained recurrent spontaneous abortion. *Medical Science Monitor*.

[12] Zhu Y., Lu H., Huo Z. (2016). MicroRNA-16 inhibits feto-maternal angiogenesis and causes recurrent spontaneous abortion by targeting vascular endothelial growth factor. *Scientific Reports*.

[13] Qin W., Tang Y., Yang N., Wei X., Wu J. (2016). Potential role of circulating microRNAs as a biomarker for unexplained recurrent spontaneous abortion. *Fertility and Sterility*.

[14] Elovitz M. A., Brown A. G., Anton L., Gilstrop M., Heiser L., Bastek J. (2014). Distinct cervical microRNA profiles are present in women destined to have a preterm birth. *American Journal of Obstetrics and Gynecology*.

[15] Sanders A. P., Burris H. H., Just A. C. (2015). microRNA expression in the cervix during pregnancy is associated with length of gestation. *Epigenetics*.

[16] Hu L., Han J., Zheng F. (2014). Early second-trimester serum microRNAs as potential biomarker for nondiabetic microsomia. *BioMed Research International*.

[17] Jiang H., Wen Y., Hu L., Miao T., Zhang M., Dong J. (2015). Serum MicroRNAs as diagnostic biomarkers for macrosomia. *Reproductive Sciences*.

[18] Song G. Y., Song W. W., Han Y., Wang D., Na Q. (2013). Characterization of the role of microRNA-517a expression in low birth weight infants. *Journal of Developmental Origins of Health and Disease*.

[19] Fu G., Brkic J., Hayder H., Peng C. (2013). MicroRNAs in human placental development and pregnancy complications. *International Journal of Molecular Sciences*.

[20] Steegers E., Von Dadelszen P., Duvekot J., Pijnenborg R. (2010). Pre-eclampsia. *Lancet*.

[21] Roberts J. M., Hubel C. A. (2009). The two stage model of preeclampsia: variations on the theme. *Placenta*.

[22] American College of Obstetricians and Gynecologists (ACOG) practice bulletin (2002). Diagnosis and management of preeclampsia and eclampsia. *Obstetrics and Gynecology*.

[23] Wu L., Zhou H., Lin H. (2012). Circulating microRNAs are elevated in plasma from severe preeclamptic pregnancies. *Reproduction*.

[24] Ura B., Feriotto G., Monasta L., Bilel S., Zweyer M., Celeghini C. (2014). Potential role of circulating microRNAs as early markers of preeclampsia. *Taiwanese Journal of Obstetrics & Gynecology*.

[25] Sandrim V. C., Luizon M. R., Palei A. C., Tanus-Santos J. E., Cavalli R. C. (2016). Circulating microRNA expression profiles in pre-eclampsia: evidence of increased miR-885-5p levels. *BJOG : An International Journal of Obstetrics and Gynaecology*.

[26] Hromadnikova I., Kotlabova K., Ivankova K., Krofta L. (2017). First trimester screening of circulating C19MC microRNAs and the evaluation of their potential to predict the onset of preeclampsia and IUGR. *PLoS One*.

[27] World Health Organization (1967). *Manual of the International Statistical Classification of Diseases, Injuries and Cause of Death, 1965 Revision*.

[28] Rai R., Regan L. (2006). Recurrent miscarriage. *Lancet*.

[29] Practice Committee of the American Society for Reproductive Medicine (2012). Evaluation and treatment of recurrent pregnancy loss: a committee opinion. *Fertility and Sterility*.

[30] Jung Y. W., Jeon Y. J., Rah H. (2014). Genetic variants in microRNA machinery genes are associate with idiopathic recurrent pregnancy loss risk. *PLoS One*.

[31] Wang X., Zhao H., Li B. (2012). Evidence that miR-133a causes recurrent spontaneous abortion by reducing HLA-G expression. *Reproductive Biomedicine Online*.

[32] Goldman-Wohl D. S., Ariel I., Greenfield C., Hanoch J., Yagel S. (2000). HLA-G expression in extravillous trophoblasts is an intrinsic property of cell differentiation: a lesson learned from ectopic pregnancies. *Molecular Human Reproduction*.

[33] Cecati M., Giannubilo S. R., Emanuelli M., Tranquilli A. L., Saccucci F. (2011). HLA-G and pregnancy adverse outcomes. *Medical Hypotheses*.

[34] Dong F., Zhang Y., Xia F. (2014). Genome wide miRNA profiling of villus and decidua of recurrent spontaneous abortion patients. *Reproduction*.

[35] He X., He L., Hannon G. J. (2007). The guardian’s little helper: microRNAs in the p53 tumor suppressor network. *Cancer Research*.

[36] WHO (1977). WHO: recommended definitions, terminology and format for statistical tables related to the perinatal period and use of a new certificate for cause of perinatal deaths. Modifications recommended by FIGO as amended October 14, 1976. *Acta Obstetricia et Gynecologica Scandinavica*.

[37] Ancel P. Y., Goffinet F., EPIPAGE 2 Writing Group (2014). EPIPAGE 2: a preterm birth cohort in France in 2011. *BMC Pediatrics*.

[38] Boardman J. P., Counsell S. J., Rueckert D. (2006). Abnormal deep grey matter development following preterm birth detected using deformation-based morphometry. *NeuroImage*.

[39] Blencowe H., Cousens S., Chou D. (2013). Born too soon: the global epidemiology of 15 million preterm births. *Reproductive Health*.

[40] Trivedi S., Joachim M., McElrath T. (2012). Fetal-placental inflammation, but not adrenal activation, is associated with extreme preterm delivery. *American Journal of Obstetrics and Gynecology*.

[41] Hassan S. S., Romero R., Vidyadhari D. (2011). Vaginal progesterone reduces the rate of preterm birth in women with a sonographic short cervix: a multicenter, randomized, double-blind, placebo-controlled trial. *Ultrasound in Obstetrics & Gynecology*.

[42] Knight A. K., Smith A. K. (2016). Epigenetic biomarkers of preterm birth and its risk factors. *Genes (Basel)*.

[43] Hassan S. S., Romero R., Pineles B. (2010). MicroRNA expression profiling of the human uterine cervix after term labor and delivery. *American Journal of Obstetrics and Gynecology*.

[44] Word R. A., Li X. H., Hnat M., Carrick K. (2007). Dynamics of cervical remodeling during pregnancy and parturition: mechanisms and current concepts. *Seminars in Reproductive Medicine*.

[45] World Health Organization (1992). *International Statistical Classification of Diseases and Related Health Problems, Tenth Revision*.

[46] Kramer M. S. (1987). Determinants of low birth weight: methodological assessment and meta-analysis. *Bulletin of the World Health Organization*.

[47] United Nations Children’s Fund (2004). *Low Birthweight: Country, Regional and Global Estimates*.

[48] Ng S. K., Olog A., Spinks A. B., Cameron C. M., Searle J., McClure R. J. (2010). Risk factors and obstetric complications of large for gestational age births with adjustments for community effects: results from a new cohort study. *BMC Public Health*.

[49] Levy A., Wiznitzer A., Holcberg G., Mazor M., Sheiner E. (2010). Family history of diabetes mellitus as an independent risk factor for macrosomia and cesarean delivery. *Journal of Maternal- Fetal and Neonatal Medicine*.

[50] Boyd M. E., Usher R. H., McClean F. H. (1983). Fetal macrosomia: prediction, risks, and proposed management. *Obstetrics and Gynecology*.

[51] Wang D., Na Q., Song W. W., Song G. Y. (2014). Altered expression of miR-518b and miR-519a in the placenta is associated with low fetal birth weight. *American Journal of Perinatology*.

[52] Li J., Chen L., Tang Q. (2015). The role, mechanism and potentially novel biomarker of microRNA-17-92 cluster in macrosomia. *Scientific Reports*.

[53] Ge Q., Zhu Y., Li H., Tian F., Xie X., Bai Y. (2015). Differential expression of circulating miRNAs in maternal plasma in pregnancies with fetal macrosomia. *International Journal of Molecular Medicine*.

[54] Zhang J. T., Cai Q. Y., Ji S. S. (2016). Decreased miR-143 and increased miR-21 placental expression levels are associated with macrosomia. *Molecular Medicine Reports*.

[55] Miura K., Higashijima A., Hasegawa Y. (2015). Circulating levels of maternal plasma cell-free miR-21 are associated with maternal body mass index and neonatal birth weight. *Prenatal Diagnosis*.

[56] Li Q., Kappil M. A., Li A. (2015). Exploring the associations between microRNA expression profiles and environmental pollutants in human placenta from the National Children’s Study (NCS). *Epigenetics*.

[57] Tsamou M., Vrijens K., Madhloum N., Lefebvre W., Vanpoucke C., Nawrot T. S. (2016). Air pollution-induced placental epigenetic alterations in early life: a candidate miRNA approach. *Epigenetics*.

[58] LaRocca J., Binder A. M., McElrath T. F., Michels K. B. (2016). First-trimester urine concentrations of phthalate metabolites and phenols and placenta miRNA expression in a cohort of U.S. women. *Environmental Health Perspectives*.

[59] Moldovan L., Batte K. E., Trgovcich J., Wisler J., Marsh C. B., Piper M. (2014). Methodological challenges in utilizing miRNAs as circulating biomarkers. *Journal of Cellular and Molecular Medicine*.

[60] Chango A., Pogribny I. P. (2015). Considering maternal dietary modulators for epigenetic regulation and programming of the fetal epigenome. *Nutrients*.

